# Dibromidobis(1,10-phenanthroline-κ^2^ *N*,*N*′)cadmium(II)

**DOI:** 10.1107/S1600536809020352

**Published:** 2009-06-06

**Authors:** Yu-Hui Sun, Shu-Fen Luo, Xiu-Zhi Zhang, Zi-Yi Du

**Affiliations:** a College of Chemistry and Life Science, Gannan Normal University, Ganzhou, Jiangxi 341000, People’s Republic of China

## Abstract

The title compound, [CdBr_2_(C_12_H_8_N_2_)_2_], synthesized by the hydro­thermal reaction of Cd(CH_3_COO)_2_·2H_2_O with NaBr and 1,10-phenanthroline, has the Cd^II^ cation coordinated by two Br^−^ anions and four N atoms from two 1,10-phenanthroline ligands in a distorted octa­hedral geometry. The crystal packing is stabilized by inter­molecular π–π inter­actions with centroid–centroid distances 3.572 (1) and 3.671 (1) Å together with C—H⋯Br hydrogen bonds.

## Related literature

For other cadmium–halogen compounds with 1,10-phenanthroline (phen) as a coligand, see: Cao *et al.* (2007 ▶); Chen *et al.* (2003 ▶); Guo *et al.* (2006 ▶); He *et al.* (2005 ▶); Li *et al.* (2007 ▶); Wang *et al.* (1996 ▶); Zhang (2007 ▶). For bond-length data, see: Allen *et al.* (1987 ▶).
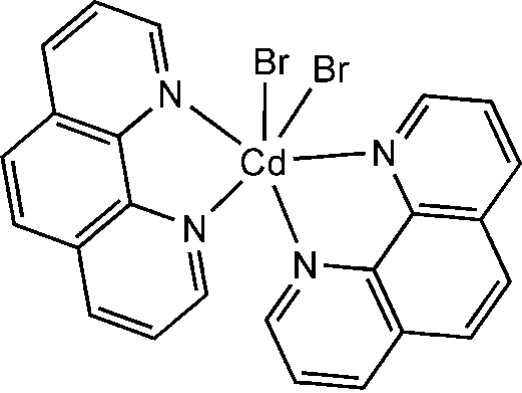


## Experimental

### 

#### Crystal data

[CdBr_2_(C_12_H_8_N_2_)_2_]
*M* *_r_* = 632.63
Triclinic, 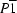

*a* = 9.3996 (2) Å
*b* = 10.1421 (3) Å
*c* = 12.8441 (3) Å
α = 78.927 (2)°
β = 81.303 (1)°
γ = 70.633 (1)°
*V* = 1128.58 (5) Å^3^
*Z* = 2
Mo *K*α radiation
μ = 4.53 mm^−1^
*T* = 296 K
0.30 × 0.28 × 0.06 mm

#### Data collection

Bruker SMART APEXII diffractometer
Absorption correction: multi-scan (*SADABS*; Bruker, 2008 ▶) *T*_min_ = 0.279, *T*_max_ = 0.762
15420 measured reflections
5656 independent reflections
4515 reflections with *I* > 2σ(*I*)
*R*_int_ = 0.026

#### Refinement

*R*[*F* ^2^ > 2σ(*F* ^2^)] = 0.037
*wR*(*F* ^2^) = 0.109
*S* = 1.03
5656 reflections
280 parameters
H-atom parameters constrained
Δρ_max_ = 1.05 e Å^−3^
Δρ_min_ = −1.16 e Å^−3^

### 

Data collection: *APEX2* (Bruker, 2008 ▶); cell refinement: *SAINT* (Bruker, 2008 ▶); data reduction: *SAINT*; program(s) used to solve structure: *SHELXS97* (Sheldrick, 2008 ▶); program(s) used to refine structure: *SHELXL97* (Sheldrick, 2008 ▶); molecular graphics: *SHELXTL* (Sheldrick, 2008 ▶); software used to prepare material for publication: *SHELXL97*.

## Supplementary Material

Crystal structure: contains datablocks I, global. DOI: 10.1107/S1600536809020352/sj2628sup1.cif

Structure factors: contains datablocks I. DOI: 10.1107/S1600536809020352/sj2628Isup2.hkl

Additional supplementary materials:  crystallographic information; 3D view; checkCIF report

## Figures and Tables

**Table 1:** Selected geometric parameters (Å, °)

Cd1—N3	2.359 (3)
Cd1—N2	2.367 (3)
Cd1—N1	2.442 (3)
Cd1—N4	2.471 (3)
Cd1—Br1	2.6249 (6)
Cd1—Br2	2.6913 (5)

**Table 2:** Hydrogen-bond geometry (Å, °)

*D*—H⋯*A*	*D*—H	H⋯*A*	*D*⋯*A*	*D*—H⋯*A*
C3—H3*A*⋯Br2^i^	0.93	2.81	3.731 (5)	171
C20—H20*A*⋯Br2^ii^	0.93	2.88	3.776 (5)	162

Symmetry codes: (i) 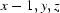; (ii) 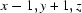.

**Fig. 1.**
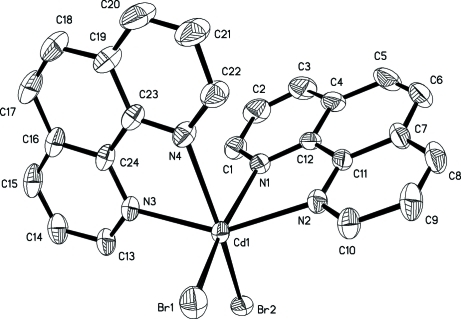
The molecular structure of compound I, with displacement ellipsoids drawn at the 30% probability level for non-H atoms.

## supplementary crystallographic information

### Comment

Recently, there have been a number of reports of cadmium-halogen complexes with 1,10-phenanthroline (phen) as a coligand (Cao *et al.*, 2007; Chen *et al.*, 2003; Guo *et al.*, 2006; He *et al.*, 2005; Li *et al.*, 2007; Wang *et al.*, 1996; Zhang, *et al.*, 2007). We have synthesized the mononuclear title  complex [CdBr_2_(phen)_2_], (I), Fig 1. The Cd^II^ cation is coordinated by two Br^-^ anions and four N atoms from two 1,10-phenanthroline ligands in a distorted octahedral geometry, Table 1. The Cd—Br and Cd—N bond lengths are in the expected ranges (Allen *et al.* 1987). It is worthy of note that compound I crystallizes in the triclinic space group *P*-1 , while the analogous mononuclear chlorido and iodido complexes [CdCl_2_(phen)_2_] (Wang *et al.*, 1996) and [CdI_2_(phen)_2_] (Cao *et al.*, 2007) crystallize in the monoclinic, *P*21/c, and orthorhombic, *P*bcn, space groups respectively.

The crystal packing is stabilized by intermolecular π–π interactions between the phen rings, with centroid-centroid distances of 3.572 (1) Å (from two adjacent C13/C14/C15/C16/C24/N3 rings) and 3.671 (1) Å (from two adjacent C7/C8/C9/C10/N2/C11 rings), and C—H···Br hydrogen bonds, Table 2.

### Experimental

A mixture of Cd(CH_3_COO)_2_.2(H_2_O) (67 mg, 0.25 mmol), NaBr (36 mg, 0.35 mmol) and 1,10-phenanthroline (69 mg, 0.35 mmol) in 10 ml distilled water was put into a Parr Teflon-lined autoclave (23 ml) and heated at 413 K for 3 days. On cooling, yellow block-shaped crystals of compound I were collected in a *ca* 55% yield based on Cd.

### Refinement

All H atoms were placed at calculated positions and refined with isotropic displacement parameters using a riding model [C—H = 0.93 Å and *U*_iso_(H) = 1.2*U*_eq_(C)].  The highest electron density peaks in the difference map, 1.05 and -1.16 Å, are close to the Cd1 and Br1 atoms, respectively.

### Crystal data

**Table:**

[CdBr_2_(C_12_H_8_N_2_)_2_]	*Z* = 2
*M**_r_* = 632.63	*F*(000) = 612
Triclinic, *P*1	*D*_x_ = 1.862 Mg m^−^^3^
Hall symbol: -P 1	Mo *K*α radiation, λ = 0.71073 Å
*a* = 9.3996 (2) Å	Cell parameters from 15420 reflections
*b* = 10.1421 (3) Å	θ = 1.6–28.4°
*c* = 12.8441 (3) Å	µ = 4.53 mm^−^^1^
α = 78.927 (2)°	*T* = 296 K
β = 81.303 (1)°	Block, yellow
γ = 70.633 (1)°	0.30 × 0.28 × 0.06 mm
*V* = 1128.58 (5) Å^3^	

### Data collection

**Table:**

Bruker SMART APEXII diffractometer	5656 independent reflections
Radiation source: fine-focus sealed tube	4515 reflections with *I* > 2σ(*I*)
graphite	*R*_int_ = 0.026
φ and ω scans	θ_max_ = 28.4°, θ_min_ = 1.6°
Absorption correction: multi-scan (*SADABS*; Bruker, 2008)	*h* = −12→12
*T*_min_ = 0.279, *T*_max_ = 0.762	*k* = −13→12
15420 measured reflections	*l* = −17→16

### Refinement

**Table:**

Refinement on *F*^2^	Primary atom site location: structure-invariant direct methods
Least-squares matrix: full	Secondary atom site location: difference Fourier map
*R*[*F*^2^ > 2σ(*F*^2^)] = 0.037	Hydrogen site location: inferred from neighbouring sites
*wR*(*F*^2^) = 0.109	H-atom parameters constrained
*S* = 1.03	*w* = 1/[σ^2^(*F*_o_^2^) + (0.0576*P*)^2^ + 1.3724*P*] where *P* = (*F*_o_^2^ + 2*F*_c_^2^)/3
5656 reflections	(Δ/σ)_max_ = 0.001
280 parameters	Δρ_max_ = 1.05 e Å^−^^3^
0 restraints	Δρ_min_ = −1.16 e Å^−^^3^

### Special details

**Table:**

Geometry. All e.s.d.'s (except the e.s.d. in the dihedral angle between two l.s. planes) are estimated using the full covariance matrix. The cell e.s.d.'s are taken into account individually in the estimation of e.s.d.'s in distances, angles and torsion angles; correlations between e.s.d.'s in cell parameters are only used when they are defined by crystal symmetry. An approximate (isotropic) treatment of cell e.s.d.'s is used for estimating e.s.d.'s involving l.s. planes.
Refinement. Refinement of *F*^2^ against ALL reflections. The weighted *R*-factor *wR* and goodness of fit *S* are based on *F*^2^, conventional *R*-factors *R* are based on *F*, with *F* set to zero for negative *F*^2^. The threshold expression of *F*^2^ > σ(*F*^2^) is used only for calculating *R*-factors(gt) *etc*. and is not relevant to the choice of reflections for refinement. *R*-factors based on *F*^2^ are statistically about twice as large as those based on *F*, and *R*- factors based on ALL data will be even larger.

### Fractional atomic coordinates and isotropic or equivalent isotropic displacement parameters (Å^2^)

**Table:**

	*x*	*y*	*z*	*U*_iso_*/*U*_eq_	
Cd1	0.55044 (3)	0.23874 (3)	0.24464 (2)	0.03912 (10)	
Br1	0.74883 (7)	0.36969 (6)	0.16057 (4)	0.06865 (16)	
Br2	0.71950 (5)	−0.02183 (4)	0.32529 (4)	0.05049 (13)	
N1	0.3404 (4)	0.1407 (3)	0.2733 (3)	0.0423 (7)	
N2	0.4886 (4)	0.2069 (4)	0.0812 (2)	0.0430 (7)	
N3	0.4726 (4)	0.3143 (4)	0.4127 (3)	0.0451 (8)	
N4	0.3237 (4)	0.4503 (4)	0.2368 (3)	0.0492 (8)	
C1	0.2693 (5)	0.1083 (5)	0.3666 (4)	0.0557 (11)	
H1A	0.3048	0.1158	0.4283	0.067*	
C2	0.1434 (6)	0.0635 (7)	0.3752 (5)	0.0752 (16)	
H2A	0.0963	0.0413	0.4423	0.090*	
C3	0.0887 (6)	0.0519 (7)	0.2884 (6)	0.0803 (17)	
H3A	0.0026	0.0240	0.2948	0.096*	
C4	0.1629 (5)	0.0824 (5)	0.1871 (5)	0.0616 (12)	
C5	0.1163 (6)	0.0697 (7)	0.0888 (6)	0.0797 (17)	
H5A	0.0316	0.0411	0.0905	0.096*	
C6	0.1928 (7)	0.0986 (6)	−0.0054 (5)	0.0750 (16)	
H6A	0.1618	0.0864	−0.0674	0.090*	
C7	0.3194 (6)	0.1471 (5)	−0.0124 (4)	0.0545 (11)	
C8	0.4023 (7)	0.1798 (5)	−0.1086 (4)	0.0663 (14)	
H8A	0.3750	0.1697	−0.1725	0.080*	
C9	0.5217 (8)	0.2258 (6)	−0.1086 (4)	0.0716 (15)	
H9A	0.5761	0.2485	−0.1724	0.086*	
C10	0.5626 (6)	0.2389 (5)	−0.0119 (3)	0.0569 (11)	
H10A	0.6445	0.2712	−0.0126	0.068*	
C11	0.3688 (5)	0.1622 (4)	0.0822 (3)	0.0427 (9)	
C12	0.2892 (4)	0.1273 (4)	0.1839 (3)	0.0436 (9)	
C13	0.5464 (6)	0.2500 (5)	0.4971 (3)	0.0561 (11)	
H13A	0.6328	0.1729	0.4895	0.067*	
C14	0.5008 (7)	0.2923 (6)	0.5971 (4)	0.0678 (14)	
H14A	0.5570	0.2452	0.6544	0.081*	
C15	0.3737 (8)	0.4027 (6)	0.6098 (4)	0.0707 (15)	
H15A	0.3421	0.4321	0.6761	0.085*	
C16	0.2903 (6)	0.4723 (5)	0.5235 (4)	0.0593 (12)	
C17	0.1551 (8)	0.5900 (6)	0.5292 (5)	0.0803 (18)	
H17A	0.1201	0.6239	0.5937	0.096*	
C18	0.0766 (8)	0.6533 (7)	0.4438 (5)	0.0835 (18)	
H18A	−0.0132	0.7272	0.4511	0.100*	
C19	0.1298 (6)	0.6082 (5)	0.3421 (4)	0.0666 (14)	
C20	0.0546 (7)	0.6714 (7)	0.2503 (5)	0.089 (2)	
H20A	−0.0360	0.7451	0.2543	0.106*	
C21	0.1138 (8)	0.6255 (7)	0.1547 (5)	0.090 (2)	
H21A	0.0651	0.6681	0.0933	0.108*	
C22	0.2493 (6)	0.5126 (5)	0.1514 (4)	0.0639 (13)	
H22A	0.2886	0.4801	0.0868	0.077*	
C23	0.2659 (5)	0.4952 (4)	0.3313 (4)	0.0505 (10)	
C24	0.3446 (5)	0.4250 (4)	0.4241 (3)	0.0467 (9)	

### Atomic displacement parameters (Å^2^)

**Table:**

	*U*^11^	*U*^22^	*U*^33^	*U*^12^	*U*^13^	*U*^23^
Cd1	0.04326 (16)	0.04325 (16)	0.03189 (14)	−0.01209 (11)	−0.00285 (10)	−0.01124 (11)
Br1	0.0775 (4)	0.0791 (4)	0.0600 (3)	−0.0404 (3)	0.0076 (3)	−0.0180 (3)
Br2	0.0427 (2)	0.0468 (2)	0.0585 (3)	−0.00646 (17)	−0.00875 (18)	−0.01007 (19)
N1	0.0415 (17)	0.0412 (17)	0.0432 (17)	−0.0114 (13)	−0.0039 (13)	−0.0065 (14)
N2	0.0506 (19)	0.0442 (18)	0.0348 (16)	−0.0137 (15)	−0.0054 (14)	−0.0081 (13)
N3	0.059 (2)	0.0428 (17)	0.0360 (16)	−0.0165 (15)	−0.0029 (14)	−0.0122 (14)
N4	0.060 (2)	0.0411 (18)	0.0392 (17)	−0.0078 (16)	−0.0006 (15)	−0.0049 (14)
C1	0.055 (3)	0.060 (3)	0.051 (2)	−0.020 (2)	0.005 (2)	−0.010 (2)
C2	0.055 (3)	0.089 (4)	0.082 (4)	−0.033 (3)	0.021 (3)	−0.016 (3)
C3	0.044 (3)	0.100 (4)	0.106 (5)	−0.036 (3)	0.004 (3)	−0.024 (4)
C4	0.040 (2)	0.066 (3)	0.084 (3)	−0.015 (2)	−0.011 (2)	−0.021 (3)
C5	0.054 (3)	0.094 (4)	0.107 (5)	−0.027 (3)	−0.034 (3)	−0.023 (4)
C6	0.070 (3)	0.079 (4)	0.084 (4)	−0.012 (3)	−0.040 (3)	−0.027 (3)
C7	0.069 (3)	0.046 (2)	0.051 (2)	−0.011 (2)	−0.027 (2)	−0.0098 (19)
C8	0.094 (4)	0.058 (3)	0.046 (2)	−0.011 (3)	−0.031 (3)	−0.011 (2)
C9	0.110 (5)	0.073 (3)	0.035 (2)	−0.033 (3)	−0.007 (2)	−0.010 (2)
C10	0.074 (3)	0.065 (3)	0.035 (2)	−0.028 (2)	−0.001 (2)	−0.0093 (19)
C11	0.045 (2)	0.0382 (19)	0.0409 (19)	−0.0033 (16)	−0.0134 (16)	−0.0078 (16)
C12	0.0322 (18)	0.042 (2)	0.055 (2)	−0.0053 (15)	−0.0084 (16)	−0.0110 (17)
C13	0.076 (3)	0.055 (3)	0.042 (2)	−0.021 (2)	−0.012 (2)	−0.0130 (19)
C14	0.102 (4)	0.070 (3)	0.041 (2)	−0.035 (3)	−0.012 (2)	−0.012 (2)
C15	0.113 (5)	0.072 (3)	0.040 (2)	−0.048 (3)	0.016 (3)	−0.024 (2)
C16	0.081 (3)	0.052 (3)	0.050 (2)	−0.028 (2)	0.015 (2)	−0.022 (2)
C17	0.098 (4)	0.070 (3)	0.068 (4)	−0.021 (3)	0.029 (3)	−0.038 (3)
C18	0.082 (4)	0.073 (4)	0.082 (4)	−0.002 (3)	0.017 (3)	−0.037 (3)
C19	0.066 (3)	0.050 (3)	0.069 (3)	−0.002 (2)	0.007 (2)	−0.014 (2)
C20	0.075 (4)	0.068 (4)	0.089 (4)	0.017 (3)	−0.004 (3)	−0.009 (3)
C21	0.089 (4)	0.071 (4)	0.074 (4)	0.023 (3)	−0.019 (3)	−0.005 (3)
C22	0.067 (3)	0.058 (3)	0.051 (3)	0.000 (2)	−0.007 (2)	−0.005 (2)
C23	0.058 (2)	0.037 (2)	0.052 (2)	−0.0112 (18)	0.0064 (19)	−0.0112 (17)
C24	0.063 (3)	0.0366 (19)	0.042 (2)	−0.0199 (18)	0.0084 (18)	−0.0128 (16)

### Geometric parameters (Å, °)

**Table:**

Cd1—N3	2.359 (3)	C7—C11	1.415 (5)
Cd1—N2	2.367 (3)	C8—C9	1.350 (9)
Cd1—N1	2.442 (3)	C8—H8A	0.9300
Cd1—N4	2.471 (3)	C9—C10	1.396 (6)
Cd1—Br1	2.6249 (6)	C9—H9A	0.9300
Cd1—Br2	2.6913 (5)	C10—H10A	0.9300
N1—C1	1.319 (5)	C11—C12	1.448 (6)
N1—C12	1.356 (5)	C13—C14	1.393 (6)
N2—C10	1.329 (5)	C13—H13A	0.9300
N2—C11	1.344 (5)	C14—C15	1.354 (8)
N3—C13	1.318 (6)	C14—H14A	0.9300
N3—C24	1.358 (5)	C15—C16	1.391 (8)
N4—C22	1.328 (6)	C15—H15A	0.9300
N4—C23	1.351 (5)	C16—C24	1.413 (6)
C1—C2	1.384 (7)	C16—C17	1.430 (8)
C1—H1A	0.9300	C17—C18	1.350 (9)
C2—C3	1.336 (9)	C17—H17A	0.9300
C2—H2A	0.9300	C18—C19	1.430 (7)
C3—C4	1.410 (8)	C18—H18A	0.9300
C3—H3A	0.9300	C19—C20	1.401 (8)
C4—C12	1.399 (6)	C19—C23	1.416 (6)
C4—C5	1.438 (8)	C20—C21	1.370 (8)
C5—C6	1.344 (9)	C20—H20A	0.9300
C5—H5A	0.9300	C21—C22	1.404 (7)
C6—C7	1.417 (8)	C21—H21A	0.9300
C6—H6A	0.9300	C22—H22A	0.9300
C7—C8	1.402 (8)	C23—C24	1.437 (6)
			
N3—Cd1—N2	149.72 (12)	C7—C8—H8A	119.9
N3—Cd1—N1	88.27 (12)	C8—C9—C10	119.4 (5)
N2—Cd1—N1	69.02 (12)	C8—C9—H9A	120.3
N3—Cd1—N4	68.75 (12)	C10—C9—H9A	120.3
N2—Cd1—N4	86.08 (12)	N2—C10—C9	122.4 (5)
N1—Cd1—N4	76.42 (12)	N2—C10—H10A	118.8
N3—Cd1—Br1	103.12 (9)	C9—C10—H10A	118.8
N2—Cd1—Br1	96.15 (9)	N2—C11—C7	122.3 (4)
N1—Cd1—Br1	163.88 (8)	N2—C11—C12	118.4 (3)
N4—Cd1—Br1	96.90 (9)	C7—C11—C12	119.3 (4)
N3—Cd1—Br2	94.02 (9)	N1—C12—C4	122.3 (4)
N2—Cd1—Br2	103.63 (8)	N1—C12—C11	118.1 (4)
N1—Cd1—Br2	85.87 (8)	C4—C12—C11	119.6 (4)
N4—Cd1—Br2	155.34 (9)	N3—C13—C14	123.0 (5)
Br1—Cd1—Br2	104.380 (19)	N3—C13—H13A	118.5
C1—N1—C12	118.6 (4)	C14—C13—H13A	118.5
C1—N1—Cd1	125.6 (3)	C15—C14—C13	119.2 (5)
C12—N1—Cd1	115.7 (3)	C15—C14—H14A	120.4
C10—N2—C11	118.8 (4)	C13—C14—H14A	120.4
C10—N2—Cd1	122.5 (3)	C14—C15—C16	120.0 (4)
C11—N2—Cd1	118.6 (2)	C14—C15—H15A	120.0
C13—N3—C24	118.6 (4)	C16—C15—H15A	120.0
C13—N3—Cd1	122.3 (3)	C15—C16—C24	117.7 (5)
C24—N3—Cd1	119.1 (3)	C15—C16—C17	123.9 (5)
C22—N4—C23	119.0 (4)	C24—C16—C17	118.3 (5)
C22—N4—Cd1	125.6 (3)	C18—C17—C16	122.0 (5)
C23—N4—Cd1	115.0 (3)	C18—C17—H17A	119.0
N1—C1—C2	121.9 (5)	C16—C17—H17A	119.0
N1—C1—H1A	119.0	C17—C18—C19	120.7 (5)
C2—C1—H1A	119.0	C17—C18—H18A	119.6
C3—C2—C1	120.8 (5)	C19—C18—H18A	119.6
C3—C2—H2A	119.6	C20—C19—C23	117.2 (5)
C1—C2—H2A	119.6	C20—C19—C18	123.3 (5)
C2—C3—C4	119.3 (5)	C23—C19—C18	119.5 (5)
C2—C3—H3A	120.3	C21—C20—C19	120.3 (5)
C4—C3—H3A	120.3	C21—C20—H20A	119.8
C12—C4—C3	117.0 (5)	C19—C20—H20A	119.8
C12—C4—C5	119.0 (5)	C20—C21—C22	118.6 (5)
C3—C4—C5	124.0 (5)	C20—C21—H21A	120.7
C6—C5—C4	121.3 (5)	C22—C21—H21A	120.7
C6—C5—H5A	119.4	N4—C22—C21	122.6 (5)
C4—C5—H5A	119.4	N4—C22—H22A	118.7
C5—C6—C7	121.5 (5)	C21—C22—H22A	118.7
C5—C6—H6A	119.3	N4—C23—C19	122.2 (4)
C7—C6—H6A	119.3	N4—C23—C24	118.9 (4)
C8—C7—C11	117.0 (5)	C19—C23—C24	119.0 (4)
C8—C7—C6	123.8 (5)	N3—C24—C16	121.5 (4)
C11—C7—C6	119.3 (5)	N3—C24—C23	118.1 (3)
C9—C8—C7	120.2 (4)	C16—C24—C23	120.4 (4)
C9—C8—H8A	119.9		

### Hydrogen-bond geometry (Å, °)

**Table:**

*D*—H···*A*	*D*—H	H···*A*	*D*···*A*	*D*—H···*A*
C3—H3A···Br2^i^	0.93	2.81	3.731 (5)	171
C20—H20A···Br2^ii^	0.93	2.88	3.776 (5)	162

Symmetry codes: (i) *x*−1, *y*, *z*; (ii) *x*−1, *y*+1, *z*.
